# Can PTSD be prevented? A novel approach to increasing physiological resilience: a pilot study

**DOI:** 10.3389/fpsyg.2023.1144302

**Published:** 2023-07-04

**Authors:** Mark Dust

**Affiliations:** ^1^School of Community and Global Health, Claremont Graduate University, Claremont, CA, United States; ^2^Department of Public Health, California State University Fullerton, Fullerton, CA, United States

**Keywords:** PTSD, resilience, heart rate variability, trauma, stress, meditation

## Abstract

Much of the U.S. adult population will experience a traumatic event at some point in their lives, resulting in about 20 million people developing posttraumatic stress disorder (PTSD) and costing over $143 billion for healthcare. The Community Resilience Model (CRM) and Mental and Emotional Self-Management (MESM) are potential novel solutions for stemming the tide of PTSD diagnoses resulting from a traumatic event. This pilot study was conducted to examine the phasic and tonic changes in cardiac vagal tone in a non-traumatized sample population (*N* = 83) after a 1-week intervention. Group comparisons were conducted between the CRM (*n* = 26), MESM (*n* = 34), and Control (*n* = 23) conditions. Participants ranged in age from 18 to 30. A phasic effect on cardiac vagal tone was found for the MESM condition within subjects but not for the CRM or control conditions. A tonic effect on cardiac vagal tone was not found within subjects amongst the three conditions. The phasic effect in the MESM condition was significantly different between itself and the CRM and control groups. No tonic effects on cardiac vagal tone were found between conditions either. These results suggest cardiac vagal tone responds to focused breathing in the moment only, but more research with larger sample sizes, longer intervention duration, and better methods to track home practice compliance are needed before one accepts the insignificant results as valid. This pilot study can serve as an introduction to the study of physiological processes that might be trainable to increase resilience in non-traumatized populations and serve as a springboard for future studies of physiological resilience to traumatic stress.

## 1. Introduction

Almost 90% of the U.S. adult population will experience a traumatic event during their lifetimes (Kilpatrick et al., 2013). Of those, approximately 6.8% will develop posttraumatic stress disorder (PTSD) (Kessler et al., 2005). To put this in perspective, the population of the U.S. in 2017 was 325.1 million people. Of those, 292.6 million will experience at least one traumatic event, 19.9 million will develop PTSD, and their symptoms may persist for their lifetimes. PTSD is a potentially debilitating anxiety disorder resulting from experiencing or witness to a traumatic event such as physical or sexual assault, natural disasters, motor vehicle accidents, and military combat. Intrusive recollections, avoidance and numbing of responsivity, and hyper-arousal characterize symptoms.

PTSD is unique, among psychological disorders, in that symptoms are tied directly to a traumatic event (Breslau, 2002). The primary symptoms have remained relatively unchanged up through the DSM-IV-TR (American Psychiatric Association, 1994), however, what constitutes a traumatic event has changed. The DSM-III defined trauma as “an event that is outside the range of usual human experience,” meaning to include combat, rape, and natural disasters (American Psychiatric Association, 1980, p. 236) while excluding more normal events like divorce, serious illness, and financial problems. The DSM-IV revised the definition of trauma to reflect research showing traumatic events to be relatively more common than previously believed. Now, traumatic events are defined as when a person “experienced, witnessed or was confronted with an event or events that involved actual or threatened death or serious injury, or a threat to the physical integrity of self or others” as well as, the individual also had a response of “intense fear, helplessness, or horror” (American Psychiatric Association, 1994).

### 1.1. Psychobiology of resilience

Resilience refers to a person’s ability to adapt successfully to traumatic, acute, or more chronic forms of adversity (Feder et al., 2009). A resilient individual demonstrates “psychobiological allostasis” (Charney, 2004). Previously it has not been possible to investigate the underlying biological processes correlated with psychobiological allostasis (Feder et al., 2009).

Differences in function, balance, and interaction of numerous hormones, neurotransmitters, and neuropeptides underlie inter-individual variance in resilience to traumatic stress (Feder et al., 2009). Excessive cortisol is associated with structural changes in the hippocampus and amygdala up to and including neuronal atrophy (Bremner, 2006), along with hypertension, immunosuppression, cardiovascular disease, and other health maladies (McEwen and Seeman, 1999). Therefore, a reduction in CRH release and adaptations in CRH receptors might promote resilience (Feder et al., 2009).

### 1.2. Psychosocial factors of resilience

A range of psychosocial factors promoting successful adaptation to stress has been identified, while the neurobiological mechanisms that underlie some of these traits are still being studied. The psychosocial factors identified are (1) facing fears and active coping; (2) optimism and positive emotions; (3) cognitive reappraisal, positive reframing, and acceptance; (4) social competence and social support; (5) purpose in life, a moral compass, meaning and spirituality (Feder et al., 2009). Positive emotions are especially interesting in that they are associated with a broadened focus of attention and decreased autonomic activity (Fredrickson, 2001). Autonomic activity is the physiological manifestation of the stress response.

### 1.3. Integrated model of resilience

The function of brain structures and circuits mediating mood and emotion are determined by a person’s psychological makeup and in turn, define their resilience to stress. Adaptation of fear, reward, emotion regulation, and or social-behavior circuits are believed to equip a resilient person’s capacity to face fears, experience positive emotions, positively reframe traumatic events and recoup the benefits of supportive friendships. “Thus, resilience is an active process, and it can be promoted by enhancing protective factors” (Feder et al., 2009). Furthermore, new intervention models might become possible through a thorough study of the neural circuitry of resilience as evidenced by recent studies demonstrating individuals modulating their brain activity using real-time fMRI-based neurofeedback (Caria et al., 2007; McCaig et al., 2011, Veit et al., 2012).

Additional support for an integrated model of resilience is found in Multiple Code Theory (MCT). Humans are complex multi-system emotional information processors possessing multiple codes, with a large number of systems that are limited in their integration and categorized into *subsymbolic, symbolic nonverbal, and symbolic verbal* codes (Bucci, 2021). Bucci (2021) differentiates subsymbolic and symbolic processing as continuous or analogic vs. discrete representational entities, respectively. Subsymbolic processing involves the subconscious processes that fire motor neurons, cause us to feel sensations, and process the world through our senses. This processing manifests as the felt sense or intuition. Symbolic processing on the other hand is based on images and words.

The referential process connecting subsymbolic and symbolic systems is key to understanding the brain body connection under investigation in this study. Images incorporating all sense modalities, as in the resourcing and resource intensification exercise described in Miller-Karas (2015) and used in this study, serve to facilitate the connection between the symbolic and nonverbal system to the subsymbolic system. This connection between systems creates emotion schemas, information from our bodies and emotions overlaid with information from past and present experiences, to make decisions on how to act, and express feelings (Bucci, 2021).

### 1.4. The polyvagal theory

Porges (1992) first proposed the use of vagal tone as an index of stress vulnerability and reactivity. He theorized the parasympathetic nervous system, via the vagus nerve, played a crucial role in mediating homeostasis and stress. By measuring cardiac vagal tone, an individual’s stress response and vulnerability to stress can be determined (Porges, 1992). Porges’s early work on vagal tone led to the development of his polyvagal theory. The polyvagal theory states each of the three primary adaptive behavioral strategies of mammals is controlled by a distinct neural circuit involving the autonomic nervous system (Porges, 2011). The three behavioral strategies are immobilization, mobilization, and social communication or social engagement. Each of these strategies corresponds with the neuroception of safety and are applied in reverse order. If the threat is determined as one that safety can be assured by facial expressions, vocalization, or listening, the myelinated vagus inhibits the influence of the sympathetic nervous system on the heart. If the threat is seen as grave harm, the sympathetic nervous system is not inhibited, and fight or flight behaviors result. If it is determined that fight or flight is no longer an option to ensure safety, the unmyelinated portion of the vagus nerve initiates behavioral shutdown, feigning death (Porges, 2011). The present study is concerned with the second and third stages of the theory.

### 1.5. The community resiliency model

The Community Resiliency Model (CRM) is a skills-based, stabilization program designed to reset the natural balance of the nervous system after a challenging experience (Miller-Karas, 2015). CRM is comprised of six skills: Tracking, Resourcing and Resource Intensification, Grounding, Gesturing, Help Now!, and Shift and Stay. The skills are intended to help people recognize signs of dysregulation in their nervous system and correct the imbalance on their own. The ability to self-regulate the nervous system has been shown to reduce distress indicators (anxiety, depression, somatic, hostility) and increase well-being indicators (relaxed, contented, somatic, friendly) in populations who are possibly suffering from cumulative trauma or undiagnosed PTSD (Miller-Karas et al., 2013) and traumatic stress symptoms from natural disasters (Citron et al., 2013). However, little is known about the mechanism by which the CRM skills facilitate these changes. This study uses participants who have not been exposed to a traumatic event or natural disaster to try and isolate the hypothesized physiological mechanism of resilience developed by the CRM skills of Tracking, and Resourcing and Resource Intensification, at what point along the trauma timeline these effects are seen and determine if a non-traumatized population will see an increase in protective factors.

Meditation and CRM could be seen as “stepchildren” from a common parent given they share some commonalities; however, they also have some clear differences. Walsh and Shapiro (2006) define meditation as:

A family of self-regulation practices that focus on training attention and awareness in order to bring mental processes under greater voluntary control and thereby foster general mental well-being and development and/or specific capacities such as calm, clarity, and concentration.

CRM and meditation both train attention and awareness but differ in that meditation is concerned with bringing mental processes under control whereas CRM is concerned with bringing physiological states under control. Mindfulness meditation develops the skill of bringing one’s attention to the present moment and awareness of perceptible mental processes, and it is believed that bringing one’s attention to these mental processes would result in a reduction in negative affect and improve coping (Grossman et al., 2004). CRM also strives to reduce negative affect and improve coping but through the mechanism of bringing one’s attention to sensations rather than perceptible mental processes. This attention to sensation appears to be a critical difference between CRM and the different forms of meditation in general. One could speculate that it is the subconscious mental processes, accessed through CRM’s attention to sensations, that have a more significant influence and control over the physiological processes that ultimately result in self-regulation of the autonomic nervous system (ANS).

### 1.6. Mental and emotional self-management

Mental and Emotional Self-Management (MESM) is a combination of the biological nervous system regulation skills taught in CRM and the practice of self-management (Hunter and Scherer, 2010) to bring awareness to internal processing and improve personal effectiveness via mindfulness meditation. In short, MESM is a mind-body approach that addresses both the physiological and psychological processes present in an individual’s excessive stress-response. The components of MESM are: education in the psychobiology of stress, the CRM skills to balance the nervous system, and breath counting skills to actively engage the parasympathetic branch of the autonomic nervous system. A pilot study of the three-week long version of MESM showed participants scored higher in mindful attention and well-being measures (Rodgers and Kettering, 2017).

## 2. Methods

### 2.1. Participants and procedure

**Figure 1:**
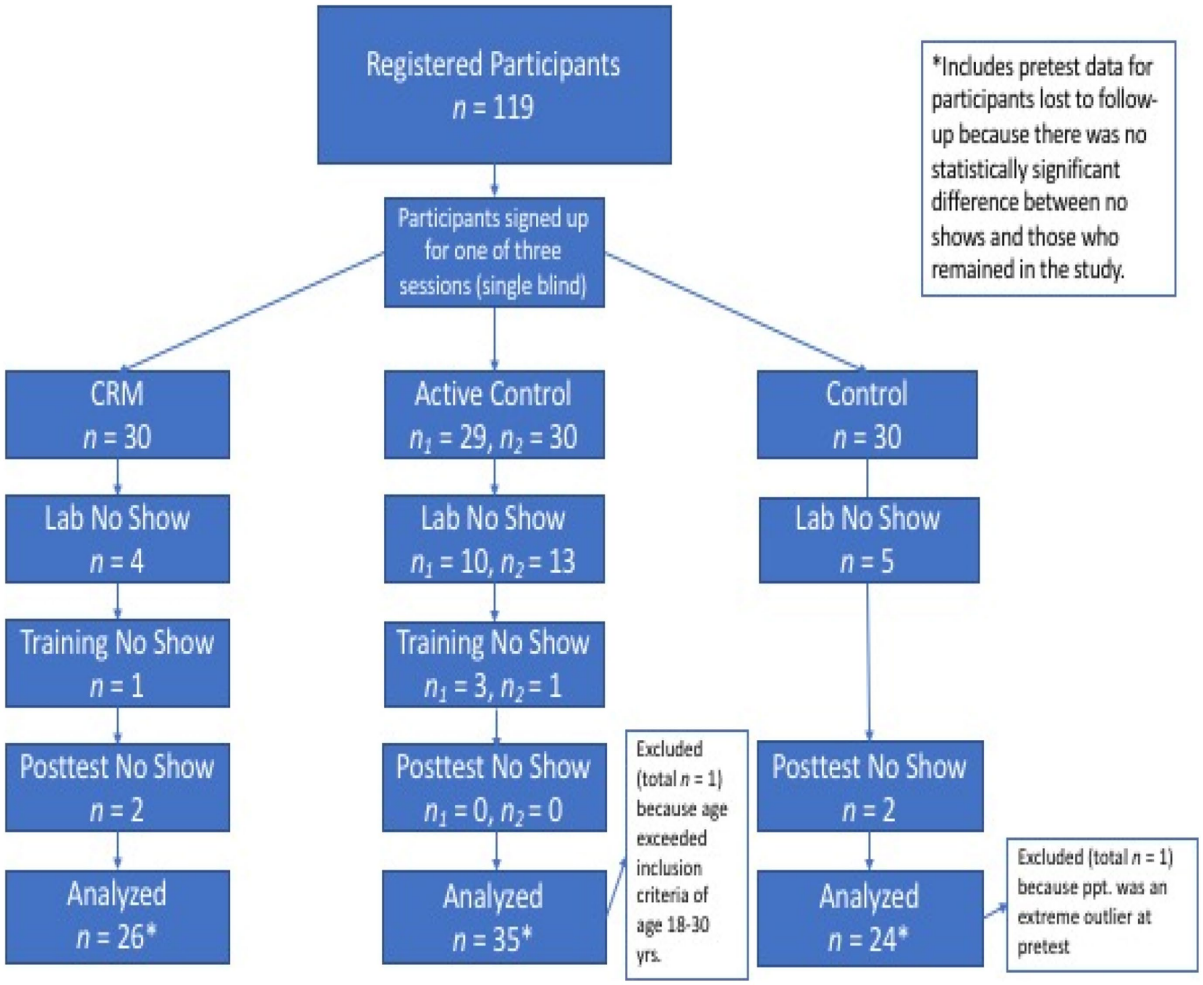
Participant randomization to condition.

A total of 119 participants from the Claremont colleges and surrounding community were recruited through mass e-mails and an existing online recruitment pool. Inclusion criteria were 18–30 years. of age, fluent in English, and own an iPhone or Android-based smartphone. Thirty-two of those failed to attend the first lab session, one did not meet the inclusion criteria, and one failed to complete the pretest survey, leaving 85 participants (48 female, 3 missing) (see Figure 1) who completed the pretest protocol (*M* = 22.5, SD = 2.83). Five participants failed to attend their designated training sessions, and four did not return for the follow-up session at the lab. Independent *t*-tests for all self-report and physiological measures revealed no statistically significant difference between the pretest means of those who dropped out and those who completed the study, nor were there significant differences between the groups at pretest (all *ps* > 0.05). Participants earned $60 for completion of the study. Study sessions were conducted at the Center for Neuroeconomics Studies (CNS) at Claremont Graduate University in Claremont, *CA.* Claremont Graduate University’s Institutional Review Board approved the study.

This pilot study aimed to recruit 30 participants for each of three study conditions, Community Resiliency Model (CRM) (*n* = 26), Mental and Emotional Self-Management (MESM) (*n* = 35), and Control (*n* = 25). The sample size was based on results from previous experiments at the CNS using video stimuli and the budget allotted to this pilot study. Potential effect sizes were unknown because of the lack of resilience research in untraumatized populations; therefore, a power analysis was not conducted *a priori*.

The recruitment email provided a link to register for involvement in the study either the first Friday, Saturday, or second Friday session and participants selected a time of their choosing. Thirty appointments, distributed at the rate of three per hour, were available each day. Participant assignment was single-blind. Participants who registered for the first Friday were placed into the CRM group. Those registered for Saturday were placed into the MESM group and the second Friday registrants were placed into the control group. Thirty-four percent of registrants for the Saturday session were no-shows, necessitating a second recruitment to meet the participant goal for the MESM group (see Figure 1).

The CRM and MESM groups each received a 3-h block of group training the following day after their lab session. The in-person training was conducted in a classroom at Claremont Graduate University. The CRM training session was facilitated by a licensed marriage and family therapist (LMFT) CRM Master Trainer with 6 years of experience delivering CRM training and a clinical psychologist CRM Master Trainer with 5 years of experience delivering CRM training. CRM skills training is usually conducted over the course of 3 days, but for this pilot study, it was condensed to 3 h with an emphasis placed on the CRM skill of resourcing. Along with learning the resourcing skill, participants were given an overview of the role the ANS plays in stress management, the Resilient Zone concept, and the CRM skills of tracking, grounding, shift and stay, and Help Now (see Supplementary Appendix B for details). After completion of training, participants downloaded the iChill app (Trauma Resource Institute, 2015) and received instructions for home practice (see Supplementary Appendix B).

There were two separate recruitments for the MESM condition (*n*_1_ = 29, *n*_2_ = 30) due to a large percentage of no-shows (*n*_1_ = 34%, *n*_2_ = 43%) for the first lab session in order to net 35 participants for this condition. The training sessions were conducted by a Ph.D. student from the Division of Behavioral & Organizational Sciences Positive Psychology program in the School of Social Science, Policy & Evaluation at Claremont Graduate University. The student is a co-developer of the MESM program that served as the basis for the training received by the participants in this condition. The in-person training was also 3 h long and delivered in a classroom at Claremont Graduate University. The training covered an introduction to social learning, basic concepts in positive psychology, the ANS’s role in stress management, and a focused attention meditation through breath counting (see Supplementary Appendix C). After completion of training, participants downloaded the Take a Break app (Maddux and Maddux, 2015) and received instructions for home practice (see Supplementary Appendix C).

The control condition received no training but was instructed to download the Elevate Brain Training application (Elevate, 2015) to their smartphone and to complete the baseline assessment for each category after leaving the lab. Participants in all three conditions were instructed to use their respective smartphone applications for approximately 15 min each day, to reinforce the skills they learned in training, until returning to the lab for follow-up testing on the seventh day.

Before consent, participants were informed that the purpose of the study was to investigate what happens in their body when they are exposed to different types of emotional stimulus. The consent form further informed participants that they would see several videos that might be distressing and cause emotional discomfort to some people. After obtaining written informed consent, participants completed a questionnaire that included demographic items and some state and trait measures. Once finished, participants were seated privately in a dimly lit room in front of a 15″ MacbookPro^©^ laptop (Apple, Inc.) equipped with headphones. All proceeding tasks were presented in MATLAB^©^ (Mathworks, Inc.), using the Psychophysics Toolbox extensions (Brainard, 1997).

After a 3-min baseline acquisition period for autonomic nervous system (ANS) measures, oddly numbered participants watched a 75-s video clip of a skydiver having a seizure during freefall followed by a 54-s video clip of an explosion that almost killed two American soldiers in Afghanistan. Even numbered participants watched a 76-s video clip of two skydiving aircraft in a mid-air collision followed by a 52-s video clip of Special Forces soldiers engaging in a firefight with insurgents. These types of videos were chosen because of their similarity to the types of traumatic events that are commonly associated with the subsequent development of PTSD. After the video stimuli participants had a 3-min recovery acquisition period for ANS measures.

Post-recovery, participants were asked to rate their emotions (results not reported here) using 12 adjectives previously used to assess empathic concern and personal distress (Batson et al., 1997). After the emotion ratings, participants completed a symmetry span task to measure working memory (results not reported here) (von Bastian et al., 2013; Stone and Towse, 2015). Lastly, participants in the CRM and MESM conditions were given directions to the location of their training session while the participants in the control condition were given instructions on how to download and use the Elevate Brain Training app for their home practice sessions along with when to return to the CNS lab for follow-up testing (see Supplementary Appendix D).

**Figure 2:**
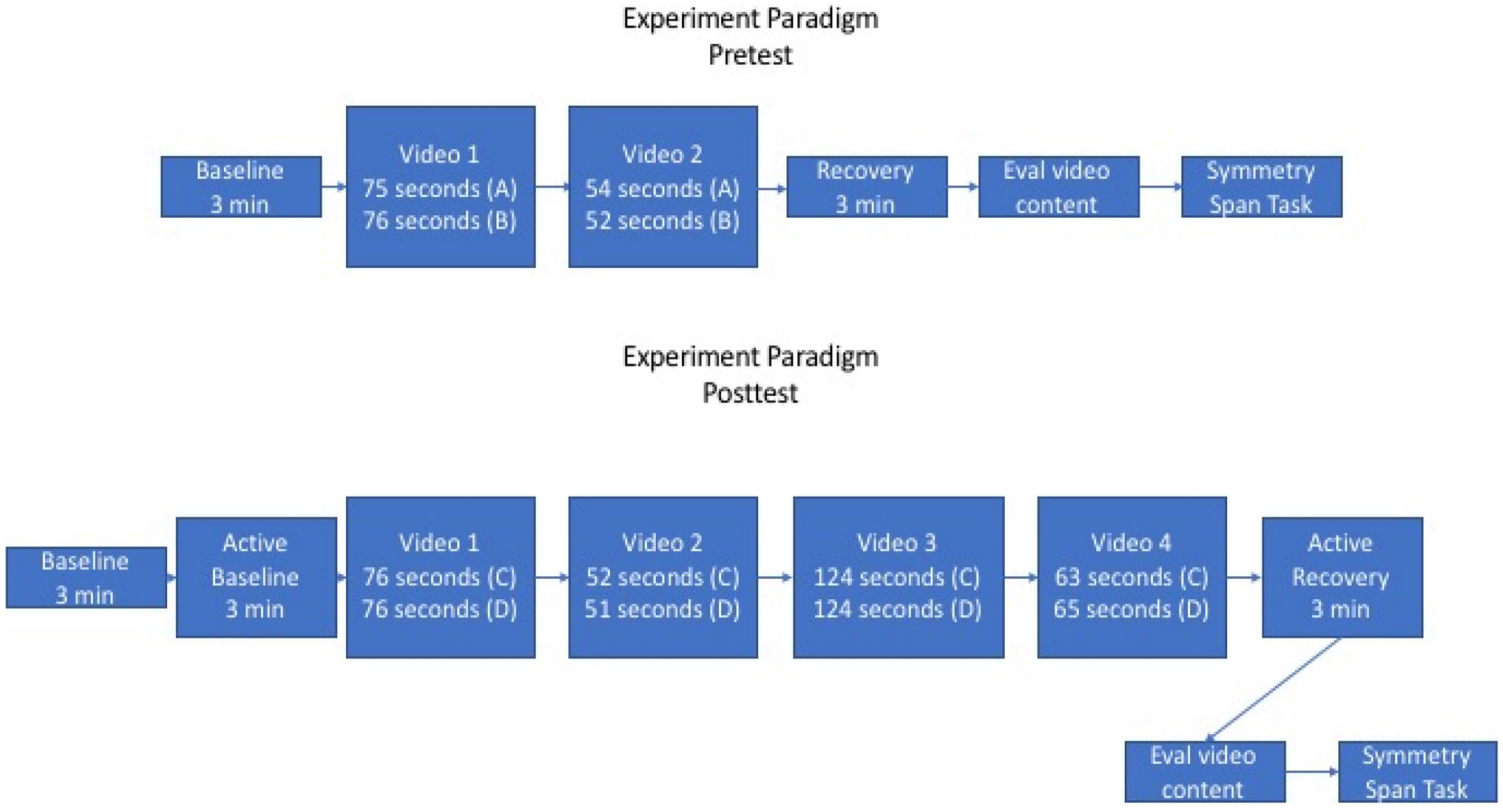
Experiment protocol for physiological measures.

Follow-up testing on the 7th day followed the same protocol as pretesting except for the addition of a 3-min active rest period immediately following the baseline ANS measures and the addition of two stimulus videos (see Figure 2). During the active rest period, participants in the CRM condition were asked to engage in the resourcing exercise, MESM participants performed the focused attention breath counting exercise, and the control participants were asked to continue to sit quietly. The addition of the two stimulus videos at posttest was done to enhance the accuracy of the physiological measures. Skin conductance (results not included here) was assessed but requires at least 2 min of data to calculate the Skin Conductance Level (SCL) (Dawson et al., 1989), and the videos at pretest barely met this requirement (129 s). The video stimuli at posttest consisted of the same two videos from pretest and two videos from a pool of four which were counterbalanced between the odd and even number participants, as well as ordered from the least to most distressing according to focus group ratings. For complete details of the experiment protocol, see Supplementary Appendix A: CRM Study Script.

### 2.2. Measures

#### 2.2.1. Autonomic measures

Cardiac (sampling rate 1 kHz) activity was collected using a Biopac MP150 data acquisition system and BioNomadix^®^ transmitters and recorded with AcqKnowledge^®^ software version 4.2 (Biopac Inc., Goleta, CA). To measure cardiac activity, participants were fitted with three disposable Ag-AgCl electrocardiogram (ECG) electrodes using a Lead(III) configuration.

Following data collection, the data were manually inspected in AcqKnowledge^®^ software version 4.2 (Biopac Inc., Goleta, CA). ECG artifacts were manually removed from the data and the data was then passed through the band-pass finite impulse response (FIR) filter, to remove both high-and low-frequency noise, and then smoothed. R-R intervals were identified and extracted from Biopac and imported into Kubios software^1^ for the derivation of heart rate variability (HRV) measures, including the high frequency (HF) component as the measure of vagal control. Linear trend components were removed from the data prior to HRV analysis. The HF power was extracted from the 0.12 to 0.40 Hz band, and then a natural logarithmic transformation was applied as suggested by Lewis et al. (2012).

## 3. Results

### 3.1. Descriptive statistics

Descriptive statistics summarizing demographic data collected at pretest are presented in Tables 1, 2.

**Table 1:** Demographics by condition.

	Condition					
	CRM (*n* = 26)		MESM (*n* = 34)		Control (*n* = 23)	
	*n*	%	*n*	%	*n*	%
Gender						
Male	13	50	12	35.3	9	40.9
Female	13	50	22	64.7	13	59.1
Race/ethnicity						
African American	3	11.5	4	11.8	1	4.5
Asian	5	19.2	5	14.7	7	31.8
Hispanic	4	15.4	3	8.8	1	4.5
Pacific Islander	1	3.8	0	0	0	0
Mixed race	1	3.8	6	17.6	2	9.1
White	12	46.2	16	47.1	11	50
Prior meditation experience						
No	7	26.9	10	29.4	6	26.1
Yes	19	73.1	24	70.6	16	69.6
Frequency of meditation practice						
Very often or always	1	3.8	2	5.9	0	0
Often	2	7.7	2	5.9	1	4.5
Sometimes	4	15.4	11	32.4	6	27.3
Rarely	10	38.5	8	23.5	9	40.9
Very rarely or never	9	34.6	11	32.4	6	27.3

**Table 2:** Descriptive statistics of pretest self-report measures by condition.

	Condition					
	CRM		MESM	Control		
Measure	*M*	SD	*M*	SD	*M*	SD
Age	21.5	2.61	22.4	2.41	23.8	3.26
SES	6.1	1.37	6.1	1.70	6.2	0.99
ACE	1.6	1.44	2.0	2.22	1.7	1.78

SES, Socioeconomic status; ACE, Adverse childhood experiences (used to screen for potential trauma exposure prior to the study.)

### 3.2. Autonomic measures—within subjects

A series of paired *t*-tests were run to determine if within-subjects effects existed amongst the conditions. The first paired-samples *t*-test determined if there was a statistically significant difference at posttest between active baseline and baseline HF HRV. This test provides evidence of a phasic response in HF HRV in response to engaging in the resourcing or breath counting exercise during the 3-m active rest period prior to the video stimuli. No outliers were detected, and all conditions were normally distributed, Shapiro-Wilk *p* > 0.05. Participants in the CRM condition (*n* = 23) did not exhibit a statistically significant difference between posttest active baseline (*M* = 6.75, SD = 0.860) and baseline (*M* = 6.83, SD = 0.921), *M* = −0.077, 95% CI [−0.398, 0.244], *t*(22) = −0.495, *p* = 0.626, *d* = 0.103. Participants in the MESM condition (*n* = 30) saw a statistically significant difference between posttest active baseline (*M* = 7.40, SD = 0.91) and baseline (*M* = 7.11, SD = 1.05), *M* = 0.285, 95% CI [0.010, 0.560], *t*(29) = 2.123, *p* = 0.042, *d* = 0.388. As expected, participants in the control condition (*n* = 23) did not demonstrate a statistically significant difference between posttest active baseline (*M* = 6.71, SD = 1.11) and baseline (*M* = 6.91, SD = 1.13), *M* = −0.202, 95% CI [−0.414, 0.011], *t*(22) = −1.97, *p* = 0.062, *d* = 0.411 (Table 3).

**Table 3:** Descriptive statistics and paired *t*-test results for posttest active baseline and posttest baseline HF HRV.

	Active baseline		Baseline			95% CI for mean			
Condition	*M*	SD	*M*	SD	*n*	Difference	*t*	df	*d*
CRM	6.75	0.86	6.83	0.92	23	−0.40, 0.24	−0.50	22	0.10
MESM	7.40	0.91	7.11	1.05	30	0.01, 0.56	2.12*	29	0.39
Control	6.71	1.11	6.91	1.13	23	−0.41, 0.01	−1.97	22	0.41

*p < 0.05.

The second set of paired *t*-tests determined if there was a statistically significant difference in HF HRV from pretest baseline to posttest baseline within each condition. This test provides evidence of a training effect for the 5 days of home practice. Three outliers in the MESM and two outliers in the control conditions were detected that are more than 1.5 box-lengths from the edge of the box in a boxplot. Inspection of their values did not reveal them to be extreme and were kept in the analysis. All conditions were normally distributed, Shapiro-Wilk *p* > 0.05. Participants in the CRM condition (*n* = 23) did not show a statistically significant difference between posttest (*M* = 6.83, SD = 0.921) and pretest baselines (*M* = 7.03, SD = 1.07), *M* = −0.20, 95% CI [−0.73, 0.34], *t*(22) = −0.766, *p* = 0.452, *d* = 0.16. Participants in the MESM condition (*n* = 29) also did not see a statistically significant difference between posttest (*M* = 7.11, SD = 1.07) and pretest baselines (*M* = 7.18, SD = 0.766), *M* = −0.074, 95% CI [−0.45, 0.30], *t*(28) = −0.41, *p* = 0.686, *d* = 0.076. Participants in the control condition (*n* = 23) also did not see a statistically significant difference between posttest (*M* = 6.91, SD = 1.13) and pretest baselines (*M* = 6.78, SD = 1.12), *M* = 0.130, 95% CI [−0.31, 0.57], *t*(22) = 0.610, *p* = 0.548, *d* = 0.13 (Table 4).

**Table 4:** Descriptive statistics and paired *t*-test results for pretest baseline to posttest baseline HF HRV.

	Posttest baseline		Pretest baseline			95% CI for mean			
Condition	*M*	SD	*M*	SD	*n*	Difference	*t*	df	*d*
CRM	6.83	0.92	7.03	1.07	23	−0.73, 0.34	−0.76	22	0.16
MESM	7.11	1.07	7.18	0.77	29	−0.45, 0.30	−0.41	28	0.08
Control	6.91	1.13	6.78	1.12	22	−0.31, 0.57	0.61	22	0.13

The third paired-samples *t*-test determined if there was a statistically significant difference in HF HRV percent change from pretest baseline to video stimulus and posttest active baseline to video stimulus within each condition. This test provides evidence for a physiological inoculation effect against distressing events. Two outliers were detected in the CRM condition that were more than 1.5 box-lengths from the edge of the box in a boxplot. Inspection of their values revealed neither one to be extreme and were kept in the analysis. All conditions were normally distributed, Shapiro-Wilk *p* > 0.05. Participants in the CRM condition (*n* = 23) did not see a statistically significant difference between posttest (*M* = −6.61, SD = 9.51) and pretest (*M* = −6.86, SD = 13.22) percent change in HF HRV values, *M* = 0.253, 95% CI [−6.13, 6.63], *t*(22) = 0.082, *p* = 0.935, *d* = 0.017. Participants in the MESM condition (*n* = 29) did not see a statistically significant difference between posttest (*M* = −4.81, SD = 8.38) and pretest (*M* = −4.58, SD = 9.98) either, *M* = −0.232, 95% CI [−5.22, 4.76], *t*(28) = −0.095, *p* = 0.925, *d* = 0.018. Participants in the control condition (*n* = 21) also did not see a statistically significant difference between posttest (*M* = −4.14, SD = 9.50) and pretest (*M* = −4.13, SD = 10.15), *M* = −0.019, 95% CI [−5.89, 5.85], *t*(20) = −0.007, *p* = 0.995, *d* = 0.002 (Table 5).

**Table 5:** Descriptive statistics and paired *t*-test results for pretest baseline to stimulus and posttest baseline to stimulus percent change in HF HRV.

	Posttest percent change		Pretest percent change			95% CI for mean			
Condition	*M*	SD	*M*	SD	*n*	Difference	*t*	df	*d*
CRM	−6.61	9.51	−6.86	13.2	23	−6.13, 6.63	0.08	21	0.02
MESM	−4.81	8.38	−4.58	9.98	29	−5.22, 4.76	−0.10	28	0.02
Control	−4.14	9.50	−4.13	10.2	21	−5.89, 5.85	−0.01	20	0.00

### 3.3. Autonomic measures—between subjects

In preparation for running a series of ANCOVA’s to determine if significant differences between the conditions exist, I investigated the correlations between demographics, self-report measures, and the autonomic measures to identify independent variables that should be controlled for in the ANCOVAs. There were no significant correlations between demographics or self-report measures and dependent variables of interest (see Tables 6, 7).

**Table 6:** Adjusted and unadjusted intervention means and variability for posttest active baseline HF HRV with posttest baseline HF HRV as a covariate.

	*n*	Unadjusted		Adjusted	
		*M*	SD	*M*	SE
CRM	23	6.75	0.86	6.85	0.13
MESM	30	7.40	0.91	7.29	0.11
Control	23	6.70	1.11	6.75	0.13

n, number of participants; M, Mean; SD, Standard Deviation; SE, Standard Error.

**Table 7:** Adjusted and unadjusted intervention means and variability for posttest baseline HF HRV with pretest baseline HF HRV as a covariate.

	*n*	Unadjusted		Adjusted	
		*M*	SD	*M*	SE
CRM	23	6.83	0.92	6.83	0.20
MESM	29	7.11	1.07	7.00	0.18
Control	23	6.91	1.13	7.05	0.20

n, number of participants; M, Mean; SD, Standard Deviation; SE, Standard Error.

An ANCOVA was run to determine if the effect of engaging in the resourcing exercise or breath counting exercise for 3 min prior to the stimulus videos would elicit an increase in HF-HRV after controlling for HF HRV during the preceding 3 min of sitting quietly. After adjustment for HF HRV at baseline, there was a statistically significant difference in HF HRV during active baseline between the conditions, *F*(2,72) = 6.01, *p* = 0.004, *η_p_*^2^ = 0.143. *Post hoc* analysis was performed with a Bonferroni adjustment. Active baseline HF HRV was statistically significantly greater in the MESM condition vs. the CRM (*M*_diff_ = 0.44, 95% CI [0.02, 0.86], *p* = 0.034) and control conditions (*M*_diff_ = 0.54, 95% CI [0.123, 0.96], *p* = 0.006).

An ANCOVA was run to determine the effect of practicing the resourcing exercise or the breath counting exercise for 15 min a day for at least 5 days on HF HRV at posttest baseline after controlling for pretest HF HRV at baseline. After adjustment for HF HRV at pretest baseline, there was not a statistically significant difference between the conditions’ HF-HRV at baseline, *F*(2,71) = 0.36, *p* = 0.696, *η_p_*^2^ = 0.010 (Table 8).

**Table 8:** Adjusted and unadjusted intervention means and variability for posttest percent change in HF HRV from active baseline to video stimulus with pretest percent change in HF HRV from baseline to video stimulus as a covariate.

	*n*	Unadjusted		Adjusted	
		*M*	SD	*M*	SE
CRM	23	−6.61	9.51	−6.46	1.90
Active control	29	−4.81	8.38	−4.86	1.69
Control	21	−4.14	9.50	−4.24	1.99

n, number of participants; M, Mean; SD, Standard Deviation; SE, Standard Error.

An ANCOVA was run to determine the effect of practicing the resourcing exercise or the breath counting exercise for 15 min a day for at least 5 days on the percent change in HF HRV from posttest active baseline to posttest video stimulus after controlling for pretest percent change in HF HRV from pretest baseline to pretest video stimulus. After adjustment for percent change in HF HRV at pretest, there was not a statistically significant difference between the conditions’ percent change in HF HRV, *F*(2,69) = 0.36, *p* = 0.702, *η_p_*^2^ = 0.010.

## 4. Discussion

The present study sought to determine if HF HRV responded to a short-term intervention designed to exercise the parasympathetic nervous system. Three sets of paired *t*-tests were used to investigate within-subject effects for each condition. Phasic HF HRV was not influenced by the CRM training when compared to baseline HF HRV values, but it was influenced by the breath counting exercise the MESM group engaged in during the 3-min active baseline period, consistent with previous research findings that show the parasympathetic can be activated via the breath. The control group did not see a significant change in HF HRV during the period of interest, as expected. The lack of change in HF HRV in the CRM group could indicate that parasympathetic tone does not respond to the resourcing exercise as hypothesized or that more than 3 min of resourcing is required to activate the PNS. Another plausible explanation is a form of testing effect but in the reverse direction. Participants could have been anticipating the viewing of distressing videos coming up next, and intrusive thoughts could have distracted them from paying attention to the sensations associated with their resource; whereas the MESM group was concentrating on the breath counting exercise and were not as aware of intrusive thoughts regarding the upcoming distressing videos.

The second analysis tested for a tonic change in HF HRV from pretest baseline to posttest baseline. None of the three conditions showed a significant difference between the time points. The most likely explanation is 15 min a day of practice for a week is not a sufficient dose to see an effect. Both experimental conditions had a minor reduction in their mean HF HRV at posttest compared to pretest. This reduction could also be related to knowing what to expect and subconsciously preparing for the stimuli.

The final within-subjects analysis of the present study investigated if there was an inoculation effect of the CRM training on the withdrawal of parasympathetic tone during the video stimulus. It was expected, if an inoculation effect existed, the percent change in HF HRV would be less at posttest than it was at pretest indicating less PNS tone withdrawal at posttest and suggesting resilience toward the stimulus (Porges, 2011). None of the conditions saw a significant difference between the percent change in HF HRV from active baseline to video stimuli at posttest when compared to pretest percent change. However, there is 0.25% less change at posttest for the CRM condition while the MESM condition had 0.23% more change at posttest and the standard deviation for the CRM condition is 14% compared to the MESM condition at 13%. Although the percent change is minor and nonsignificant it may indicate the potential for an inoculation effect for CRM to exist considering the MESM group had an increase in PNS tone (indicated by the increase in HF HRV during active baseline) before the posttest video stimuli yet had a slightly greater percent change at posttest than pretest indicating more PNS tone withdrawal for the MESM condition.

The increase in HF HRV within the MESM condition at posttest active baseline resulted in a significant difference, after controlling for posttest baseline HF HRV, between it and the CRM and control conditions. This difference indicates a phasic response to the breath counting exercise on vagal tone and could be a potential malleable protective factor. There were no significant differences between the three conditions on tonic vagal tone nor the percent change in HF HRV during the video stimulus; although the CRM condition did have a slight improvement in percent change after adjustment for pretest percent change while the MESM group had a very slight increase after adjustment. While the changes are not statistically significant either within or between groups, the direction of the change could be indicative of a potential inoculation effect for the CRM condition. The lessening of the withdrawal of vagal tone during the video stimulus corresponds with Porges’s Polyvagal Theory (Porges, 2011). The hypothesized priming of the ANS by the breath counting exercise prior to the video stimulus does not appear to provide an inoculation effect as evidenced by the increase in PNS tone withdrawal. More research is needed on this potential effect and caution is urged before interpreting these results as anything more than a trend in a particular direction.

### 4.1. Limitations

Although the present study revealed only one significant within-subjects physiological result, this line of research needs further study. This pilot study was underpowered and only had an approximately 50% chance of detecting an effect. The budget did not allow for more than 90 participants and spreading those across three conditions further weakened the power of the study. Participant’s buy-in for the CRM and MESM conditions could have been affected as well. Several of the post-test exit surveys commented the training seemed to be more appropriate for subjects who have been exposed to a traumatic event or are suffering from traumatic stress symptoms and found it hard to relate the skills to their current states of depression and or anxiety. Participants in both treatment conditions mentioned they found it hard to find the time to practice the skills, had been in a fight with their significant other during the week, or already did most of this stuff on their own. The timing of the study could also have compromised the results. Training and data collection occurred late in the spring semester and students were most likely preparing for final exams to occur within a week or two of posttest. The short time frame also impacted on the delivery of the CRM training. CRM is usually taught over a three-day period with multiple opportunities to practice the skills. Our trainers were forced to compress 3 days of training into one 3-h block on a Saturday and the opportunities to practice the skills and receive feedback were not optimal.

## 5. Conclusion

Although little evidence was found for the efficacy of using the CRM or MESM skills to bolster physiological resiliency in this pilot study, when the study limitations are considered, there are definite areas for improvement in the design and delivery of the training and home practice compliance. The positive changes seen in the MESM group’s physiological measures indicate that PNS tone can be actively bolstered and the slight trend toward an inoculation effect during video stimulus in the CRM group warrants future study to better understand the mechanism that might be at work. Future studies incorporating CRM training as a primary or secondary prevention method for non-traumatized populations should consider re-working the training materials to incorporate a perspective of prevention or one of building resilience just in case something happens to them.

## Data availability statement

The raw data supporting the conclusions of this article will be made available by the authors, without undue reservation.

## Ethics statement

The studies involving human participants were reviewed and approved by Institutional Review Board of Claremont Graduate University. The patients/participants provided their written informed consent to participate in this study.

## Author contributions

MD is responsible for the conception, design, data collection, analysis, interpretation, drafting, revising, and approval of the manuscript.

## Funding

Funding for this research was provided by an intramural grant from the Claremont Graduate University Research Initiatives Fund.

## Conflict of interest

The author declares that the research was conducted in the absence of any commercial or financial relationships that could be construed as a potential conflict of interest.

## Publisher’s note

All claims expressed in this article are solely those of the authors and do not necessarily represent those of their affiliated organizations, or those of the publisher, the editors and the reviewers. Any product that may be evaluated in this article, or claim that may be made by its manufacturer, is not guaranteed or endorsed by the publisher.

## Footnotes

^1^ http://kubios.uef.fi
