# Supplementary material for: Can PTSD be prevented? A novel approach to increasing physiological resilience: a pilot study
Source: Front Psychol. 2023 Jul 4;14:1144302. doi: 10.3389/fpsyg.2023.1144302 (PMC10353430; doi:10.3389/fpsyg.2023.1144302)
Supplement: Supplementary file 1 [file Data_Sheet_1.docx]

Supplementary Material

Can PTSD be Prevented? A Novel Approach to Increasing Physiological Resilience: A Pilot Study

Mark Dust, Ph.D.*

*** Correspondence:** Mark Dust, Ph.D.: [mdust@fullerton.edu](mailto:mdust@fullerton.edu)

1. **Appendix A**
2. Pretest Video Stimuli Odd Number Participants
   1. Skydiver Seizure: <https://youtu.be/55QUQHm2B5A>
   2. Explosion Two Soldiers: <https://youtu.be/r0km94VCyek>
3. Pretest Video Stimuli Even Number Participants
   1. Skydive Plane Collision: <https://youtu.be/7p6hqMnsLFY>
   2. Special Forces Insurgents: <https://youtu.be/_vbfo6U4faw>
4. Posttest Video Stimuli Odd Number Participants
   1. Skydiver Seizure: <https://youtu.be/55QUQHm2B5A>
   2. Special Forces Insurgents: <https://youtu.be/_vbfo6U4faw>
   3. Skydive Plane Collision: <https://youtu.be/7p6hqMnsLFY>
   4. I-94 Car Pileup: <https://youtu.be/F79TcVsP54w>
5. Posttest Video Stimuli Even Number Participants
   1. Skydiver Seizure: <https://youtu.be/55QUQHm2B5A>
   2. Explosion Two Soldiers: <https://youtu.be/r0km94VCyek>
   3. Skydive Plane Collision: <https://youtu.be/7p6hqMnsLFY>
   4. I-94 Car Pileup: <https://youtu.be/F79TcVsP54w>

Focus Group Video Ratings

N = 11

Questions:

1. This clip is stressful to watch.
2. I found it hard to wind down after watching this clip.
3. I felt my heart speed up while watching this clip.
4. I feel like my body is affected by watching this clip.

Scored on a Likert Scale 1-5 (Strongly disagree – Strongly agree)

Eight videos were rated:

1. I-94 car pileup (4.2)
2. Skydiving plane collision (3.9)
3. Special Forces insurgents (3.7)
4. Explosion with two soldiers (3.7)
5. Skydiver seizure (3.6)
6. Avalanche (3.6)
7. Squid attack (3.4)
8. Shark cage attack (3.4)
9. **Appendix B**
10. **Study Script**

- Goal N= 90, 3 sessions with N=3 every 25 min
- Subject ID code: C1-90
- 4 RAs show 20 mins prior to scheduled time

**Lab Schedule**:

Control **TBA**

Treatment **Apr 24** 9:30am-12:30pm; 2-5pm **May 1** 9:30am-12:30pm; 2-5pm

Active Control **Apr 25** 9:30am-12:30pm; 2-5pm **May 2** 9:30am-12:30pm; 2-5pm

**Training Schedule:**

Control None

Treatment **Apr 25** 8:30am-11:30am; 12-3pm

Active Control **Apr 26** 8:30am- 11:30am

**Materials needed:**

- Cash = $5,400 ($60 x 90) Day 7
- **Sign-in sheet** (3)
- 60 treatment **consent forms**
- 30 control **consent forms**
- 90 pay **receipts**
- 5 clipboards
- 90 saliva kits
- 360 EDA electrodes
- 540 ECG electrodes
- **90 SES Ladder survey**
- **90 Day 3-6 Handouts** (include home practice journal)

**PREP & TASKS**

- RA1: ready consent forms on clipboards, receipts, sign in sheet
- RA2-4: turn on Biopac, computers, get electrodes

**DAY 1 PROCEDURES**

0:00 Check-in

- N= 3 check-in and have study explained both verbally and in Consent form.
- Ss earns $60 in this study for completing study on day 7
- RAs explain exp during check-in (come in 3 separate days for experiment and active control groups, complete smartphone tasks for 4 days; or come in on 2 separate days for no training control group and complete smartphone tasks for 4 days)

0:05 Consent and Survey

- Instruct to do survey 1 (**http://tinyurl.com/CRMpresurvey**)
- At the end of survey 1 we need the control group to download Elevate and complete the initial setup. (8-10) min

0:25 Testing Room

- Ss gets assigned to RA based on gender
- Ss asked to go wash hands
- Hold Ss until room is ready (RA1 can fit with electrodes if same gender and there is no back up at check-in)
- RA 1 hands off Ss to matching gender RA 2-4 when room is available
- RA 2-4 fits Ss with electrodes if not already fitted
  - Have Ss take seat and explain the next steps of the protocol
    - quiet period where you sit still
    - videos containing emotional content
    - second quiet period
    - evaluating video content
    - symmetry span task
  - Check for good Biopac signal
  - Start Psychopy
    - ODD participants start **CRM_A.psyexp**
    - EVEN participants start **CRM_B.psyexp**
  - Close divider curtain and instruct Ss to relax and remain as motionless as possible while baseline data is collected (3 min)
  - Computer instructs Ss to begin video presentation (2 min)
  - Computer instructs Ss to relax and remain as motionless as possible for a 3 minute break before the next task.
  - Remove from Psychopy (ESC to exit) and Instruct Ss to begin the next task (symmetry span)
  - Click “Start” in the Tatool - Symmetry Span window
    - Enter 5 digit Ss ID#, i.e. C45 is entered 00045
    - Describe task to Ss (instructions are small and hard to read)
      - Ss will be presented with a blue square on a 4x4 matrix and must remember it’s position within the matrix.
      - Ss will be presented with a pattern and must determine if it is symmetrical or non-symmetrical
      - If it is symmetrical, press the key with the RED dots
      - It it is not symmetrical, press the key with the BLUE dots
    - Ss is tasked to remember the position and the order in which the blue squares appeared.

Ss will be prompted to click the position and the order in which the squares appeared.

When the program is preparing the next trial, a big blue and grey icon that looks like a “Play” button appears on screen, there is no need to click it, the program progresses on its own.

Instruct Ss to inform you when the task is complete.

- - Once complete
    - Save the data file
    - Remove Biopac connections
    - Instruct Ss on electrode removal procedure
    - Escort Ss to RA 1 for exit instructions
  - Prepare room for next Ss

0:45 Check-out

(Once Ss completes testing room tasks)

- RA1 instructs on next steps for participation including date/time of Training for experiment and active control groups.
- No training control group is reminded to complete the Elevate brain training exercises once a day for five consecutive days before returning to the lab on day 7.
- Ss are dismissed.

**DAY 2 PROCEDURES**

0:00 Check-in

- N= 3 check-in
- Ss earns $60 in this study for completing study at this visit

0:05 Survey

- Instruct to do post survey (**http://tinyurl.com/CRMpostsurvey**)

0:15 Testing Room

- Ss gets assigned to RA based on gender
- Ss asked to go wash hands
- Hold Ss until room is ready (RA1 can fit with electrodes if same gender and there is no back up at check-in)
- RA 1 hands off Ss to matching gender RA 2-4 when room is available
- RA 2-4 fits Ss with electrodes if not already fitted
  - Have Ss take seat and explain the next steps of the protocol
    - quiet period where you sit still
    - videos containing emotional content
    - second quiet period
    - **saliva collection**
    - evaluating video content
    - symmetry span task
  - Check for good Biopac signal
  - Start Psychopy
    - ODD participants start **CRM_C.psyexp**
    - EVEN participants start **CRM_D.psyexp**
  - Close divider curtain and instruct Ss to relax and remain as motionless as possible while baseline data is collected (3 min)
  - Computer instructs Ss to begin video presentation (5 min)
  - Computer instructs Ss to relax and remain as motionless as possible for a 3 minute break before the next task.
  - **Collect Saliva**
  - Remove from Psychopy (ESC to exit) and Instruct Ss to begin the next task (symmetry span)
  - Once complete
    - Save the data file
    - Remove Biopac connections
    - Instruct Ss on electrode removal procedure
    - Escort Ss to RA 1 for exit instructions
  - Prepare room for next Ss

0:45 Check-out

(Once Ss completes testing room tasks)

- Ss are compensated and dismissed.

1. **Appendix C**
2. **TRM Wellness Skills: The Community Resiliency Model**

**By Elaine Miller-Karas**

**Elainemk27@mac.com**

This material cannot be copied without the express written permission of the author.

1. **Module One**
2. **Learning the Wellness Skills**
3. **Overview**
4. Introduction
5. Introduction to the Community Resiliency Model
6. A brief and simple introduction to:
   1. Resilient Zone
   2. Stuck in the low/high zones
   3. Autonomic Nervous System
7. The Resilient Zone Scale
8. The CRM skills of Tracking, Resourcing, and Resource Intensification will be defined and practiced.
9. **Objectives**

The participant will be able to describe:

1. The Resilient Zone
2. Being stuck in high/low zones
3. The accelerator and brake of the nervous system
4. One or more ways to track the nervous system
5. Resourcing and Resource Intensification
6. One objective of the Community Resiliency Model
7. **Module One: Step-by-Step**
8. Introduce yourself and explain why you are presenting this model. Share with the participant a bit about yourself and how you became interested in CRM. To engage the participant, use one of the following resourcing questions:
   1. What or who helps you the most now?
   2. What or who is helping you get through?
9. Introduction to Community Resiliency Model (CRM):

*Script: What Is CRM*

1. CRM is based upon current research about the brain.
2. CRM is a method that will help you understand why you may feel anxious all the time or why you may feel depressed and disconnected or why you may feel both.
3. You will learn the CRM skills and how to apply them to your daily life. The skills of CRM will help you interrupt reactions that may be troubling to you and which may have developed since ___________.
4. It is important for you to know that our nervous systems are designed to be reset and to come back to balance.
5. CRM skills can help your body and mind return to balance. I will help you learn the skills and teach you how to track sensations that can help you feel more calm and resourced.
6. Explain Resilient Zone

*Script: Resilient Zone*

1. It is important for you to know that no matter what your symptoms were as you walked in today, the skills you will learn have a capacity to help you feel better in both body and mind. The human body and mind have an amazing capacity to heal.
2. The nervous system can be likened to a computer. It receives information and then processes that information and produces a result. It regulates the activities of the body and mind. There are some terms I want you to become familiar with today—the first is your Resilient Zone.
3. Every person has a Resilient Zone. When we are in our Resilient Zones, we can handle the daily life stresses without getting bumped out of our Resilient Zone.
4. People say that they feel like themselves again when they are in their Resilient Zone. Some people after a traumatic experience like being in combat may not be able to sense their Resilient Zone.
5. One of our goals is to help you find your Resilient Zone again or to expand the existing capacity of your Resilient Zone.

As you describe the normal wave of the Resilient Zone, you can draw it on a white board or poster paper.

1. Explain Resilient Zone Scale

*Script: Resilient Zone Scale*

1. The Resilient Zone Scale can be used to track whether you are in your Resilient Zone or whether you are stuck in the high or low zones. One of the goals is to expand or deepen your Resilient Zone so that you experience it more often. Your ability to handle life stresses and the triggers that may occur because of past traumas increases as you expand your zone.
2. Paying attention to whether you are in your Resilient Zone or bumped out in the high or low zone is one way to gain greater awareness. Once your start regularly tracking your nervous system, you can be more aware of when you are in your Resilient Zone and also start paying attention to what knocks you out and, most importantly, learn the wellness skills to help you bounce back into your Resilient Zone.
3. On a scale of 1-10, write down the number in the boxes below that describes whether you are in your Resilient Zone. The red yardstick shows that 1-3 represents Stuck on Low, 4-6 represents your Resilient Zone, and 8-10 represents Stuck on High. As you learn the skills, the goal is to be within your Resilient Zone more often.
4. Explain “Stuck in High or Low Zones”

*Script: Stuck on High/Low*

1. Many experiences can bump us out of our Resilient Zones (traffic, waiting for appointments, family members, etc.). When you get bumped out of your Resilient Zone by stressful or traumatic life events or reminders of those events (the thunderbolt), look at what happens to your Resilient Zone.
2. You can get stuck in the high or low zones or go back and forth between the two. Let’s look at the symptoms that go along with being stuck in the high or low zones.
3. The good news is that CRM skills will help you get back into your Resilient Zone when you get bumped out.
4. In CRM, we believe that your reactions are common reactions to extraordinary life experiences that happen after a traumatic event. The skills will help you rebalance your body and mind and come back into your Resilient Zone.
5. These reactions are about your biology not about mental weakness.

(SHOW THE GRAPHIC FROM THE CUE CARDS OR WORKBOOK)

1. Explain the Brake and Accelerator of the Nervous System

*Script: Autonomic Nervous System*

1. When we get stuck, our nervous system is not in balance. There are two parts of the nervous system: the “accelerator” or the sympathetic nervous system and the “brake,” or the parasympathetic nervous system.
2. Being what we call stuck in the high zone is when the accelerator is down to the floor and the body can react with a fast heart rate, rapid, shallow breathing, etc.,
3. When we are in our Resilient Zone we can have different sensory experiences - our breathing may slow down and becomes deeper, our heart rate may slow down and our muscles relax. We also may track our heart rate and breathing being faster like when we experience joy…but the sensation is pleasant. The Resilient Zone is about our vitality and both biological reactions could occur and you are still within the zone.
4. It is important to remember this is a system that is hard-wired in all of us. CRM skills can help you get this system back into balance. The nervous system can be reset.
5. Introduce CRM skills of Tracking, Resourcing, and Resource Intensification

*Script: For Tracking*

1. The first three skills we will learn today are Tracking, Resourcing, and Resource Intensification.
2. In order to begin to bring your nervous system back into balance, you must first learn how to track your inner sensations. It is not uncommon for people to be very aware of discomfort and pain. However, many do not pay attention to the comfortable or neutral sensations within the body and mind.
3. To reestablish your inner balance, it is necessary to pay attention to sensations of comfort and/or neutrality within your body. This stimulates the part of your nervous system that helps you rest and digest, (the parasympathetic NS) which helps you get back into your Resilient Zone.
4. Paying attention means bringing your awareness to the sensations associated with comfort (deeper breathing, slower heart rate, muscle relaxation, spaciousness, etc.).
5. One of CRM’s goals is to help you be the best tracker of your own nervous system.
6. As we start, I will help you observe by bringing attention to a deeper breath and any changes in body posture that may indicate you are bouncing back into your Resilient Zone.
7. I will also ask you questions like “What are you noticing now?” to bring your attention to sensation change.

*Script: Explaining Resourcing and Resource Intensification*

1. A resource can be anything that helps a person feel better.
2. It can be something the person likes about himself, a positive memory, a person, place, animal, spiritual guide, faith, or anything that provides peace, joy or calm.
3. Can you tell me one resource that comes to mind?
4. Resource intensification means providing more detail about your resource. We need to have more descriptions of your resource to override the sensations connected to discomfort. Can you tell me three or more details about the resource?
5. So, now, I would like to invite you to notice what is happening inside as you think about the resource and your descriptions of the resource.
6. Draw your attention to sensations that are pleasant or neutral to you.
7. Notice what is happening to your breath, heart rate, muscle tension. Stay with that for a few moments.
8. What did you notice?

*CRM Alert: What if someone becomes distressed or cannot feel anything? If this happens, you can try one of the following:*

1. *Ask the person if he can touch the chair and ask him if he can feel the texture of the chair.*
2. *Ask for permission to touch the person’s shoulder or hand and ask him if he can track the sensations.*
3. *Resources can have many natures. For example, if the resource is the person’s mother and she has died. The person can remember her fondly but also experience sadness at her passing. After honoring the sadness, the practitioner can gently shift attention back to the mother as a resource by asking the group member what qualities he loved about his mother and then track the sensations connected to those memories.*
4. **Module Two**
5. **Overview**
6. Introduce Grounding
7. **Objectives**

The Group member will be able to describe:

1. the Autonomic Nervous System
2. Grounding and how to apply this skill to activities of daily living
3. **Module Two: Step-by-Step**
4. Introduce Skill #4, GROUNDING

*Script:*

1. Grounding is the direct contact of the body with something that provides support to the body. You can ground by sitting in a chair, standing against a wall, walking and paying attention to how your feet make contact to the ground, lying down on the floor or on a bed.

Grounding can help you stay in the present moment to experience physical and emotional safety. When you are grounding, you are in the present moment and you will not be thinking about or sensing things in the past or the future.

As you read the script, make sure to give enough time between each bullet point as it takes time for sensations to develop.

1. Find a comfortable position; take your time. As you find a comfortable position, bring your attention to a part of your body that feels more comfortable or neutral.
2. Notice how that part of your body is making contact with the chair, sofa, wall, floor, etc.
3. Now bring your attention to how your back is making contact with the chair, sofa, wall, etc. Slowly guide yourself down your body and notice how each part is making contact with a solid surface, and lastly, notice your feet making contact with the ground.
4. Notice the sensations that are more pleasant to you or neutral within your body. Take your time.
5. If you become aware of uncomfortable sensations, bring attention to places that feel neutral or better. Take your time.
6. As you bring your attention to the contact of your body to the chair/floor/wall/bed, notice your breathing, heart rate, muscle relaxation, etc.
7. As we get ready to end, slowly scan your body and bring your attention to all sensations that are pleasant or neutral.

*CRM ALERT: For some individuals who have experienced high intensity physical trauma, bringing attention to sensations in the present moment through Grounding can trigger trauma sensations. If this is the case, you can shift the person’s attention back to a resource or to the Help Now strategies.*

1. **Module Three**
2. **Overview**
3. Discuss the organizing principles of the brain, highlighting the survival brain and the importance of sensation
4. Discuss the defensive responses “tend and befriend,” “fight, flight and freeze”
5. Discuss sensations of release
6. **Objectives**

The Group member will be able to:

1. Describe the three parts of the brain (Thinking, Feeling, Survival)
2. **Module Three: Step-by-Step**
3. Education re: the Organizing principles of the brain

*Script:*

1. The brain is part of the nervous system. There are many complex ways to describe the brain. For our purposes, we will talk about the brain having three parts. The three parts are the “thinking” brain, the “emotional” brain and the “survival” brain.
2. The “Thinking” Brain is your center for executive functioning and when you are using your “Thinking” brain you can make better decisions. The “Emotional” brain assesses risk and sounds an alarm when you are in danger, and the “Survival” brain triggers the defensive responses that can help you survive if you are threatened.
3. When faced with danger that is perceived as life threatening, the human body goes into automatic defensive response. This happens without thinking, and comes from the Survival Brain. This part of the brain does not respond to “talking.” It is about keeping us alive, as the brain is designed to help us live if threatened.
4. The survival brain responds to sensation. This is why many of our skills will help you “sense” changes in your body in order to reset your nervous system so that your body and mind can come back into balance.

1. Education about Survival Responses

*Script:*

1. The survival responses can be triggered by traumatic experiences. The body has a natural defensive system. It first may try to “tend and befriend”—to reach out to the threat (women use this strategy more than men). If the threat is great enough, “tend and befriend” may be bypassed and a person may go into fight or flight when perceiving an “inescapable attack.”
2. When the body goes into fight and flight, the accelerator of the body is activated (the Sympathetic Nervous System). Sometimes people get stuck there. They can get stuck on “high.” What are the physical reactions of being stuck on high?
3. If a person gets stuck here, his body does not know the threat is over and cues that remind him of the event can be triggered. It can feel like the traumatic event is happening all the time. (The practitioner may want to give an example.) Watch for activation and shift to resourcing or grounding as needed.
4. CRM skills help the body know the threat is over by putting on the brakes (the parasympathetic nervous system). What are the reactions when the brake is on?
5. Education about the survival response of “Freeze,” also known as the 1000-yard stare.

*Script*

- When the traumatic experience overwhelms the nervous system and the person cannot get away or fight, and at the same time is terrified, the freeze response can result. In the military, the freeze response is known as the “1000-yard stare.” People who experience a freeze response have a greater chance of developing more serious reactions as time goes by. The freeze response is hard-wired within the body. It is not a choice. It happens without thinking and it can result in decreasing or increasing the chances of survival. However, the freeze response can occur and leave a negative imprint. Without understanding the automatic nature of the freeze response, people can make faulty assumptions. The faulty assumptions can include thoughts such as, “I am weak” or “I am a coward.”
- A history of traumatic experiences increases the chance of a freeze response. If a person was abused as a child, he has a greater chance of experiencing a freeze response as an adult when experiencing something traumatic. The reason for this is that children, when abused, often respond by freezing. A history of freezing increases the risk of that automatic defensive response being activated during subsequent traumatic experiences.

1. Education about Release sensations

*Script:*

- As you track your Nervous System, you will notice your Resilient Zone more often. As your body comes into balance, you may notice what is called Nervous System Release.
- Nervous System Release is a biological process that happens automatically when your body releases stuck energy. This can cause sensations that can be unsettling unless you understand their purpose. If you notice any of the sensations of release, just let them happen.
- Release sensations are a way that your Nervous System rebalances itself even though the sensations may feel strange. If the release sensations are too strong, you can consciously stop them and notice the urge to release, without allowing all the energy to discharge at once. This can be a way of slowly letting the body release.
- Release sensations include tingling, trembling, warmth, cooling down, burping, clearing of the throat, and more.

1. Family members. Orient the group member that it can be helpful for significant supportive family members to learn about CRM and learn about the biological perspective of your symptoms.

Depending on how your group is put together, you can invite the family member for an educational session with regard to the model. You could say, if you would like to bring your _________ to the next session, it can be very helpful for her to learn about the concepts we will be discussing next week.

1. **Module Four**
2. **Learning the Wellness Skills**
3. **Overview**
4. Discuss the Appraisal System of the body and brain
5. Review the Resilient Zone Scale
6. Introduce Skill 5: HELP NOW!
7. **Objectives**

The Group member will be able to:

1. Describe the purpose of the appraisal system of the body
2. Describe Skill 4 and Skill 5—Gesturing and HELP NOW!
3. **Module Four: Step-by-Step**
4. Introduce content area: Appraisal System, Fast and Slow System and Resilient Zone.

*Script:*

1. We will talk about a part of your brain called the amygdala. We will call the amygdala part of your body’s appraisal system. It scans the environment for anything that is different or new. This part remembers negative experiences so that it can warn you if there is danger based on your life experience. Some people call it your personal “alarm system.”
2. The amygdala decides whether something in the environment is safe or dangerous and it determines whether to trigger two different systems in your body: the fast system or the slow System. The fast system reacts quickly and triggers the fight and flight response when it detects danger. It is automatic and it helps you survive dangerous or life-threatening situations. When the appraisal system does not detect threat, the slow system is triggered and then your brain can take its time to respond to a situation and make plans.
3. The problem is when someone has experienced trauma, the appraisal system can lose its ability to tell the difference between situations that are dangerous and those that are safe. want to help you with the CRM skills that can help your appraisal system tell the difference between a match and a forest fire. If there are many triggers in the environment, it can be a real problem and your reactions may be much bigger and potentially lead to all sorts of problems with family, friends, and the law.
4. The good news is that the CRM skills can help reduce the power of the triggers and in some cases they may go away altogether. As you apply the CRM skills in your daily life, you will find that you are more often in your Resilient Zone. When we are in our zones, we think better and also can manage our emotions better.
5. As we have discussed these new concepts, do you have questions? Can you think of times that your body has been in the fast system? The slow system?
6. Introduce Gesturing and spontaneous Movements

*Script:*

1. Gesturing refers to:
   - A movement, usually of the body or limbs, which expresses or emphasizes an idea, sentiment, or attitude.
   - The use of motions of the limbs or body as a means of expression and the practice of focusing on those that is self-soothing.
2. There are different kinds of movements and they are usually just below your conscious awareness. If you start paying attention to gestures that are self-calming, your gestures can help you stay within your Resilient Zone.
3. The following represent the types of gestures that others have found helpful to pay attention to:
   - Self-calming: bring comfort and safety
   - Release: represent the body releasing sensations of stress or trauma
   - Universal: represent wholeness, spiritual beliefs, or deep personal meaning
   - Joyful and Powerful: represent well-being
   - Protective: movements of the hand, leg, and whole body that we do spontaneously to protect ourselves.
4. Exercise:
   - Take 5 seconds to think about a self-soothing gesture…count 1, 2, 3, 4, 5, and then make the gesture.
   - Take 5 seconds to think about a gesture of joy or confidence…count 1, 2, 3, 4, 5 and then make the gesture.
   - As you do your gesture of joy, notice what happens inside…
   - You can begin bringing awareness to gestures that are self-calming and even intentionally make a gesture that helps you as you move through your daily activities.
5. Introduce HELP NOW!

*Script:*

- When people have experienced challenging events such as combat zone trauma, sexual abuse, or other difficult situations, triggers can happen “out of the blue” and suddenly you are amped up or the opposite, you feel depressed and disconnected, which we call ramped down.
- HELP NOW, strategies can help. It can also be helpful to educate your close friends and family members on these strategies because sometimes we need help if we get really stuck.
- Any person who has experienced trauma or stress can get bumped out of his Resilient Zone. If the bodily sensation is too overwhelming, the following suggestions may help a person get back into their Resilient Zone.
  - Open your eyes if having a tendency to shut
  - Drink a glass of water
  - Look around the room and pay attention to anything that attracts your attention
  - Name six colors you see in the room or outside
  - Notice the temperature in the room
  - Notice the sounds around you
  - Count backwards from 20 to 0
  - Walk and pay attention to the movement in your arms and legs and how your feet are making contact with the ground
  - Push your hands against the wall or door slowly and notice your muscles pushing. While leaning against the wall face forward, slowly push your back against the wall.
  - Think of a sound or smell that is pleasant to you
- Not every one of the strategies will help if you are way out of your Resilient Zone. You may find one more helpful than others. The more your practice, the better you will be able to manage these sensations. It is also okay to ask for help if you are being knocked out a lot.

1. **Module Five**
2. **Learning the Wellness Skills**
3. **Overview**

Module 5 introduces the concept of “memory capsules” as a way to understand how memory can be stored as a multisensory experience and how easily it can be to be triggered by external or internal sensations. The scaffolding for the learning of the Community Resiliency Model continues with the learning of “Shift and Stay.”

1. **Objectives**

The Group member will be able to describe:

1. The concept of memory capsules
2. “Shift and Stay”
3. **Module Five: Step-by-Step**
4. Introduce content area: Memory Capsules

*Script:*

1. When people have experienced challenging events including being in a combat zone where a person may have seen, heard, smelled, and felt difficult things, the brain may not remember the experience as a “story.”
2. The various parts of the experience can be stored as pieces of sensations and images and when triggered, it can feel as if the experience is happening in the present moment. Dr. Robert Scaer refers to this form of memory as “Memory Capsules.”
3. “Memory Capsules” are triggered by a whole variety of cues or triggers that can unexpectedly result in a memory capsule releasing some of the contents into your body and mind.
4. You may not have any idea why you are experiencing sensations of distress. Since the contents are not all neatly connected, they can emerge as emotion, a sensation like nausea or shaking, or other sensory images like sounds and smells, intrusive thoughts, etc. There may not be a memory connected with the sensations.
5. By using CRM skills, you can help to reduce the power of the memory capsule and the triggers that cause unexpected and unpleasant sensations that make you feel uneasy, fearful, or out of control.
6. Discussion of content

*Script:*

1. As we have discussed the concept of memory capsules, do you have questions?
2. Have you had experiences that you think might be a memory capsule being triggered?
3. As we talk about this, it will be important to track your sensations.
4. If you notice that you are starting to be bumped out of your Resilient Zone, you can ground and resource to restore balance to your nervous system.
5. I also may suggest that we pause while you relate an experience and bring your awareness to the present moment through a grounding or resourcing exercise.
6. Content Area: Introduce Shift and Stay

*Script:*

1. Shift and Stay means shifting your attention away from something unpleasant to something pleasant or at least neutral, and then staying there.
2. If you feel distressed, you can:
   - Move your attention to a place in the body that is more comfortable, calmer, or neutral, or
   - Use one of your resources, and then notice the places inside that are calmer or neutral, or
   - Bring attention to how your body is making contact with the chair, sofa, ground, and notice the places that are more pleasant or neutral inside
   - Make a self-calming gesture and notice the sensations
   - Bring your attention to one of the HELP NOW strategies
   - Track what happens inside as you stay focused on the more positive or neutral sensations
   - Bring your attention to the whole body and track all the changes. Stay with that for a few minutes.
3. We’ll practice Shift and Stay” now.
   - Choose a resource from your list
   - You can open or close your eyes; whichever is more comfortable for you.
   - Now bring the resource to mind and bring in the details of the resource.
   - Notice the sensations.
   - Now think about a future daily routine or incident that is mildly upsetting to you.
   - As you think about that mildly upsetting daily routine, shift your awareness to the image of the resource and notice the sensations associated with the resource. Stay with the sensations that you notice when you focus on your resource.
   - As you bring in the resource, notice the changes that happen inside.
4. Do the best you can. If you notice tension arising or uncomfortable feelings or sensations let me know so I can help you shift back by staying with resource sensations.

*Note to practitioners: As you did in previous sessions, track closely so that you can help if you see any signs of difficulty. Help shift out of traumatic sensations by intensifying the resource by asking sensory and image-oriented questions (example: Let yourself take in all the details of your resource…notice all the parts that attract your attention….is there a pleasant sound or smell? What is the temperature?)*

1. Discussion about Exercise and Closing Session

*Script:*

1. We’ve got to wrap up for today but I want to emphasize again the importance of practicing the CRM skills you are learning in order to spend more time sensing your Resilient Zone.
2. The more that you practice the CRM skills, the more you will experience your Resilient Zone.
3. This can make a very big difference in managing yourself when you get very agitated.
4. The skill of “Shift and Stay” will help you return to your Resilient Zone. People often say that they feel more in charge of their sensations rather than the sensations being in charge of them.
5. **Homework-Module Five**
6. Practice the skill of Resourcing at least once when you are already feeling calm, just to remind yourself where the resource sensations are in your body. Again this week try Resourcing as you are about to go to sleep. Using this skill at bedtime may help you sleep better.
7. As you go through each day every now and then check in to see what how resilient you feel. If your Resilient Zone scale is low, bring one of your TRM skills in to help.
8. If you notice frustration, tension, or any other unpleasant symptoms during the week, use the skill of “Shift and Stay” to bring your attention to one of the resources you identified, to Grounding, to a self-soothing gesture, or to a place in the body that feels neutral or more pleasant and track the sensations associated with the skill you are using. Pay particular attention to where in your body you notice these sensations. Stay with the pleasant or neutral sensations until you come back into your Resilient Zone.

**Module Six**

1. How he is incorporating all six skills into his activities of daily living
2. **Module Six: Step-by-Step**
3. CRM Skills Exercise and Future template:

*Script:*

1. Choose a resource from your resource list
2. As you bring the resource to mind, think about three descriptors about your resource
3. Notice all the sensations that are pleasant or neutral
4. Now, think about a future daily routine that is mildly upsetting to you.
5. As you think about that mildly upsetting daily routine, shift your awareness to one of the following:
   - The resource
   - Grounding
   - A pleasant or neutral sensation within the body
   - A self-calming gesture
   - Help Now strategies
6. Discussion about exercise and Closure of Module

*Script:*

1. I want to emphasize again the importance of practicing the CRM skills you are learning in order to reset your nervous system and be aware of your Resilient Zone.
2. The more that you practice the CRM skills; the more you will experience your Resilient Zone. This can make a very big difference in managing yourself when you get very agitated.
3. The skill of Help Now! will help you come back into your Resilient Zone. This is another skill that can help you be in charge of your symptoms.
4. If you notice frustration, tension, or any other unpleasant symptoms during the week, use the skill of “Shift and Stay” and stay with the pleasant or neutral sensations until you come back into your Resilient Zone.
5. Use Help Now! strategies if you need to.
6. Practice the skills using the iChill app everyday until you return. You can login to iChill by using the app on your smart phone. Listen to one of the skills each morning. When you open the iChill app, our tracker will keep login that you are using the app.
7. SEE YOU NEXT WEEK!
8. **Appendix D**
9. **iChill Home Practice Instructions**

**Initial Set-up**

Please download the iChill app from the iTunes store:

<https://itunes.apple.com/us/app/ichill/id403527676?mt=8>

or the Google Play store for your Android device:

<https://play.google.com/store/apps/details?id=com.tritrc.ichill>

The first time you open the app it will ask you for your participant ID#. Please enter the ID# given to you during your visit to the lab yesterday. ***You will not be able to go back and enter it once you have passed this screen!***

The home screen is what you will see next. Feel free to explore all of the options here, but the button labeled “Skills” is the one you will be using most. Tap the “Skills” button and you will see the four skills you learned during the training: Tracking, Resourcing, Grounding, and Shift & Stay. Your 20 minutes of daily practice will be spent on these skills.

Please fill out the daily home practice log at the end of each day. There is no penalty for missing a day or only practicing one of the skills. Just note how much time you spent each day doing the skills practice. Also please be honest in recording your time. Again, there is no penalty for not doing the full 20 minutes each day, but it will be easier to track how effective your practice is for you if you honestly tell us how many minutes you practiced each day.

**Days 3-6**

- Listen to the **Resourcing** and **Grounding** skills **at least once** per day until you return to the lab at the end of the week.
  1. Choose a time during the day when you can commit to practicing both skills for about 20 minutes without distraction.
  2. You are also welcome to listen to the Shift & Stay skill as part of your 20 minutes of daily practice or whenever you feel like you are being bumped out of your Resilience Zone.
- Please fill out the daily home practice log at the end of each day.

**Day 7**

- Return to the Center for Neuroeconomics Studies lab, at your designated appointment time on **Friday May 1**, for the conclusion of the experiment and to receive your payment. Please bring your home practice log with you at this time.

1. **Appendix E**
2. **MESM Training**

Mental and emotional self-management (MESM) training at Claremont Graduate University, Claremont, CA is an exercise to build your skills of focus and concentration. Your trainer is Rhonda Kay Rodgers, Ph.D. student, from the School of Behavioral and Organizational Sciences, Claremont Graduate University (CGU). Ms. Rodgers will provide a lecture on and training in self-management practices and how these practices can assist in building both personal and professional well-being.

 "As much as we pump iron and we run to build our strength up, we need to build our mental strength up... so we can focus... so we can be in concert with one another."

**Phil Jackson, former coach of the LA Lakers and Chicago Bulls, with 11 NBA championships as head coach**

“You are about to embark upon a course typically reserved for top-level executives, athletes, and MBA students. The full version of this course is lengthy and expensive, however this training is critical for individuals at all levels of any organization. This training is provided in a condensed version, which means that you will do the bulk of the work outside of class time. As with all things in life your success will increase with practice and persistence.” Rhonda Kay Rodgers, MBA

**Course Format**

Lab visit (Day 1)

Mental and Emotional Self-Management Training (Day 2)

Daily exercises (Day 3-6)

Return to lab (Day 7)

**Weekly Exercise**

**Guided Meditations (days 2 through 6)**

Meditation is a key part of your training. A free downloadable app will assist you in this aspect of training. For the next week, your dedicated practice will be at least **once a day for 13 minutes. Please use the 13-minute stress relief meditation** available through the links below**.** You are also welcome to use the 7-minute work break meditation, but this is not required for the study.

Please access the guided meditation for iphone:

<https://itunes.apple.com/us/app/take-break!-guided-meditations/id453857236?mt=8>

or Android:

<https://play.google.com/store/apps/details?id=com.meditationoasis.takeabreak>

**Daily Log**

You will also keep a log of the actual time you spend in meditation each day. Your instructor will provide you with a template to fill out and turn in along with your weekly assignment.

**Home Practice Survey**

At the end of day 6 (before you return to the lab), you will be asked to fill out a home practice survey. These are a few questions designed to learn more about your experiences with home practice. Please feel free to add any additional comments that you would like to share about your experiences over the past week.

**In Class Training Outline**

1. An introduction to exemplars in the field of self-management which serve as social models for learning (Bandura, 1971), and to the possibility that humans have the ability to influence processes in the mind to change behavioral patterns
2. An introduction to basic concepts in positive psychology: flow and the control of psychic energy (Csikszentmihalyi, 1990) , positivity (Fredrickson, 2009), fixed vs. growth mindset (Dweck, 2006), judger vs. learner orientation (Adams, 2009), and the workings of the adaptive unconscious (Wilson, 2002), etc.
3. An overview of current neuroscience research on mind-wandering, meditation, and neuroplasticity
4. An introduction to the evolutionary biology of the brain (survival, procreation, and pattern recognition)
5. An introduction to the behavioral neuroscience of amygdala response to stressors and non-conscious reactivity
6. Instruction on focused attention (FA) meditation through breath counting
7. Instruction in the process of body scanning (noticing sensations in the body)
8. An in-class exercise to practice the breath counting (FA) meditation.
9. **Appendix F**
10. **Elevate Brain Training Home Practice Instructions**

**Day 1: Initial App Set-up**

Download the free Elevate app from iTunes for your iPhone:

<https://itunes.apple.com/us/app/elevate-brain-training/id875063456?mt=8>

and Google Play for your Android device:

<https://play.google.com/store/apps/details?id=com.wonder>

Enter your name, age and email address. (The research staff will not have access to this information).

Select all of the training goals offered, a normal weekly goal of 5 sessions, and turn training reminders on.

Complete the initial set-up testing and record your starting Elevate Proficiency Quotient (EPQ) scores on the home training log.

The five EPQ categories are:

Writing

Listening

Speaking

Reading

Math

**Days 2-6**

Once each day, complete the 3 daily challenges (total task time 5-8 minutes).

Please do not play more games then the three presented per day during the time you are participating in the study.

At the end of each day’s training session, select the Performance tab and record your Elevate Proficiency Quotient for each of the five categories.

**Day 7**

Return to the Center for Neuroeconomic Studies lab, at your appointment time, for the conclusion of the experiment and to receive your payment. Please bring your home practice log and home practice survey with you on day 7.

**
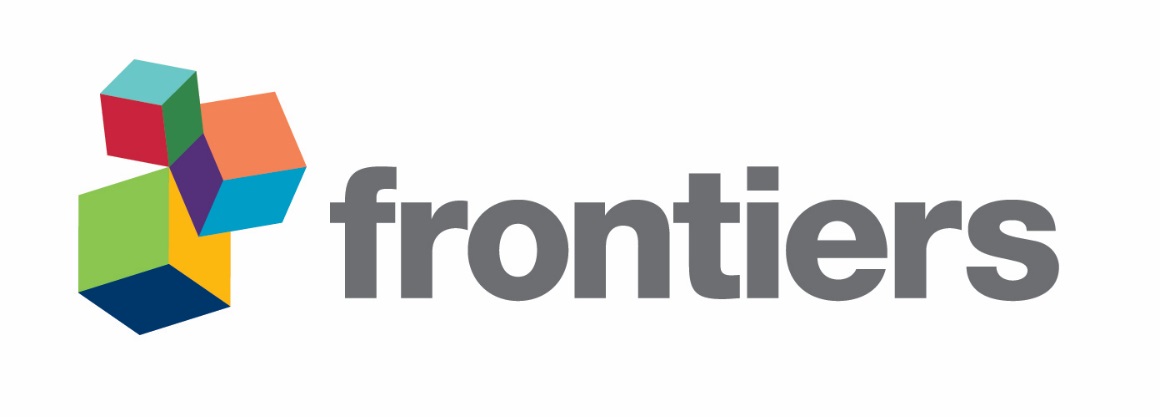
**
